# Relationship of back muscle and knee extensors with the compensatory mechanism of sagittal alignment in a community-dwelling elderly population

**DOI:** 10.1038/s41598-021-82015-8

**Published:** 2021-01-26

**Authors:** Shinji Takahashi, Masatoshi Hoshino, Shoichiro Ohyama, Yusuke Hori, Akito Yabu, Akio Kobayashi, Tadao Tsujio, Shiro Kotake, Hiroaki Nakamura

**Affiliations:** 1 Department of Orthopaedic Surgery, Osaka City University Graduate School of Medicine, 1-4-3 Asahi-machi, Abeno-ku, Osaka, 545-8585 Japan; 2 Department of Orthopaedic Surgery, Shiraniwa Hospital, Nara, Japan; 3 Kotake Orthopaedic Clinic, Nara, Japan

**Keywords:** Risk factors, Epidemiology, Disability, Disease prevention

## Abstract

Compensatory mechanisms, such as a decrease in thoracic spine kyphosis and posterior tilting or rotation of the pelvis, aim to achieve optimal alignment of the spine. However, the effect of muscle strength on these compensatory mechanisms has not been elucidated. This study aimed to investigate the impact of back muscle and lower extremity strength on compensatory mechanisms in elderly people. Overall, 409 community-dwelling elderly participants (164 men, 245 women) were included. Age, disc degeneration, and 2 or more vertebral fractures showed a significant increase of risk for sagittal vertical axis (SVA) deterioration. Conversely, stronger back, hip flexor, and knee extensor muscles reduced the risk for SVA deterioration. To investigate the association of each muscle’s strength with compensatory mechanisms, 162 subjects with pelvic incidence-lumbar lordosis > 10° were selected. The linear regression model for thoracic kyphosis demonstrated a negative correlation with back muscle strength and positive correlation with vertebral fracture. The regression analysis for pelvic tilt demonstrated a positive correlation with knee extensor strength. Back, hip flexor, and knee extensor muscle strength were associated with sagittal spinal alignment. Back muscle strength was important for the decrease in thoracic kyphosis, and knee extensor strength was associated with pelvic tilt.

## Introduction

Sagittal alignment is important to quality of life (QOL) in elderly people. Sagittal malalignment leads to back pain, gait disturbance, fatigue, and gastrointestinal symptoms^1–6^. Corrective surgery is often performed to treat the symptoms of spinal deformity, despite the substantial risk of complications^7^. Several factors, including age, osteoporotic vertebral fractures, disc degeneration, and muscle weakness, have been reported as causes of sagittal malalignment^8–13^. Osteoporotic vertebral fractures are common in elderly people, with a prevalence of 27.1% and 53.0% in female patients in their 70 s and those aged ≥ 80 years, respectively^14^. Furthermore, disc degeneration, especially degeneration of the lower lumbar disc, is very common in the elderly^15^. These factors irreversibly influence the sagittal alignment in elderly people. Conversely, strong back extensors are associated with a decrease in thoracic kyphosis (TK) and an increase in lumbar lordosis (LL) and sacral inclination and have a positive correlation with physical activity^16^.

Harmony between the lumbar spine and pelvis is critical for adequate spinal sagittal alignment. When imbalances occur, the sagittal vertical axis (SVA) moves forward. Compensatory mechanisms, including a decrease in TK, posterior tilting or rotation of the pelvis, hip extension, and even knee flexion, aim for optimal alignment of the spine, with the objective of preserving the appropriate position of the gravity line and horizontal gaze^10,17^. Jackson et al. demonstrated that subjects with low LL experienced back pain if the SVA did not improve owing to compensatory mechanisms^5^. The significant correlation between pelvic tilt (PT) and walking disability indicates that high PT does not permit effective ambulation^4^. Moreover, high PT with high SVA worsens pain and disability^4^. Therefore, compensatory mechanisms are essential to maintain function and reduce symptoms, even in subjects with high PT.

The association between back muscle strength and TK has been established^18–20^. However, the effect of muscle strength on PT has not been elucidated. Therefore, this study investigated the impact of back and lower extremity muscle strength on the compensatory mechanisms of elderly people.

## Methods

From June 2016 to January 2017, we recruited participants, aged 65 or more, from Ikoma city, Nara, Japan, via a community notice in the city. The inclusion criteria were ability to visit the hospital for the survey, ability to walk independently, and a willingness to participate in this survey. Self-administered questionnaires were sent to 458 volunteers. After written informed consent was obtained, all participants (164 men and 245 women) were included in this study. We evaluated the Charlson Comorbidity Index (CCI) and the Oswestry Disability Index (ODI).

The study protocol was approved by the Institutional Review Board (Ethical Committee of Osaka City University Graduate School of Medicine, No. 3484). All procedures performed in studies involving human participants were in accordance with the ethical standards of the institutional and/or national research committee and with the 1964 Declaration of Helsinki and its later amendments or comparable ethical standards.

### Image data

Sagittal alignment parameters and pelvic parameters were measured on a standing whole spine radiograph using Surgimap Spine (Nemaris, Inc, New York, NY, USA). The sagittal alignment parameters included SVA, TK (T5–T12), and LL (L1–S1). The pelvic parameters included PT and pelvic incidence (PI). The global sagittal alignment was typically assessed by calculating the SVA, which is the offset between the posterior corner of the sacrum and the vertical line passing through the vertebral body of C7. Mismatch between pelvic incidence and lumbar lordosis (PI-LL) was also evaluated.

Whole-spine magnetic resonance imaging (MRI) was also performed for all participants using the Philips Achieva 3.0 Quasar (Phillips, Androver, MA, USA). The imaging protocol included sagittal T2-weighted fast spin echo (FSE) (repetition time [TR]: 6320 ms/echo, echo time [TE]: 120 ms, field of view [FOV]: 270 × 270 mm) and axial T2-weighted FSE (TR: 4000 ms/echo, TE: 120 ms, FOV: 180 × 180 mm). Sagittal T2-weighted images were used to assess the intervertebral space from C2/3 to L5/S1. The degree of disc degeneration on MRI for each disc was classified into five grades based on Pfirrmann's classification system^21^ from L1/2 to L5/S1. Osteoporotic vertebral fractures (OVF) were also diagnosed using MRI.

Weight-bearing anteroposterior knee radiographs were evaluated by the Kellgren–Lawrence (K–L) grading system. Grade 2 or higher was defined as osteoarthritis. If the grades of the right and left knee joints in one individual were different, the more severe grade was used. Measurements of bone mineral density were performed by dual-energy X-ray absorptiometry at the femoral neck.

### Muscle strength

Back muscle strength was determined by the maximal isometric strength of the trunk muscles in a standing position with 30°-lumbar flexion via a digital back muscle strength meter (T.K.K. 5402, Takei Co., Japan) consisting of a handlebar attached to a floor-mounted load cell^1^. A hand-held dynamometer (µTas F-1, Anima Corp., Tokyo, Japan) was used to obtain the isometric measure of the hip flexor and knee extensor muscle strength for both lower extremities. Hip flexor strength measurement was conducted with the participant in a sitting position and the hip/knee joint flexed to 90°^22^; the participants were asked to place their hands at the armpits to stabilize the trunk. The hand-held dynamometer sensor was placed on the front of the most distal part of the thigh and just proximal to the ankle on the front of the lower leg (on the distal tibia and anterior tibial muscle tendon) for hip flexor and knee extensor measurements, respectively^23^.

### Muscle mass

Muscle mass was measured using a bioelectrical impedance analysis machine (MC780A, TANITA, Japan)^24^. Appendicular skeletal muscle mass (ASM) was calculated as the sum of the skeletal muscle masses of the arms and legs. The skeletal muscle mass index (SMI) was defined as the ASM divided by height in meters squared (ASM/height^2^).

### Statistical analysis

The χ^2^ test or Fisher exact test were used for categorical variables, and the analysis of variance or Kruskal–Wallis test were used for continuous variables.

SVA was divided into three categories (< 40 mm, 40–95 mm, and > 95 mm) based on Schwab’s classification^25^ to determine the risk factors using a proportional odds model. The risk factors included age, sex, body mass index (BMI), CCI, number of OVF (0, 1, 2, or more), disc degeneration, back muscle strength (per 1 kg) or back muscle strength (> 60 kg in male participants and > 35 kg in female participants), hip flexor strength (per 1 kg), knee extensor strength (per 1 kg), and osteoarthritis of the hip and knee (≥ K–L grade 2). The receiver operating characteristic (ROC) curve was used to investigate the area under the curve (AUC) of the back muscle strength of participants with an SVA > 95 mm. The cut-off for back muscle strength was calculated using the ROC curve. Hip flexor and knee extensor strength were excluded in this model because the AUC for both was less than 0.7 (data not shown).

Patients with PI-LL mismatch > 10° were excluded to facilitate investigation into the association of muscle strength with compensatory mechanisms. A multivariate linear regression model was used to investigate the association of each muscle’s strength with the SVA, PT, and TK. PT and TK were representative parameters of compensatory mechanisms^17^. The linear regression model was adjusted for age, sex, BMI, total disc degeneration, PI, LL, and PT or TK. PI was included in this model because the shape of the pelvis appeared to influence the type of degenerative disease observed; patients with disc lesions were characterized by a normal or low PI with a straight spine, while patients with degenerative spondylolisthesis demonstrated pronounced spinal curves with high PI [32].

To investigate the effect of compensatory mechanisms, we compared ODI according to high/low PT and SVA (Group 1: PT < 20° and SVA < 40 mm, Group 2: PT ≥ 20° and SVA < 40 mm, Group 3: PT < 20° and SVA ≥ 40 mm, Group 4: PT ≥ 20° and SVA ≥ 40 mm) using analysis of variance. The Tukey test was used for post-hoc analysis. All p values were two-sided, and p < 0.05 was considered significant. All analyses were performed using SAS version 9.4 (SAS Institute, Cary, NC, USA).

## Results

**Figure 1:**
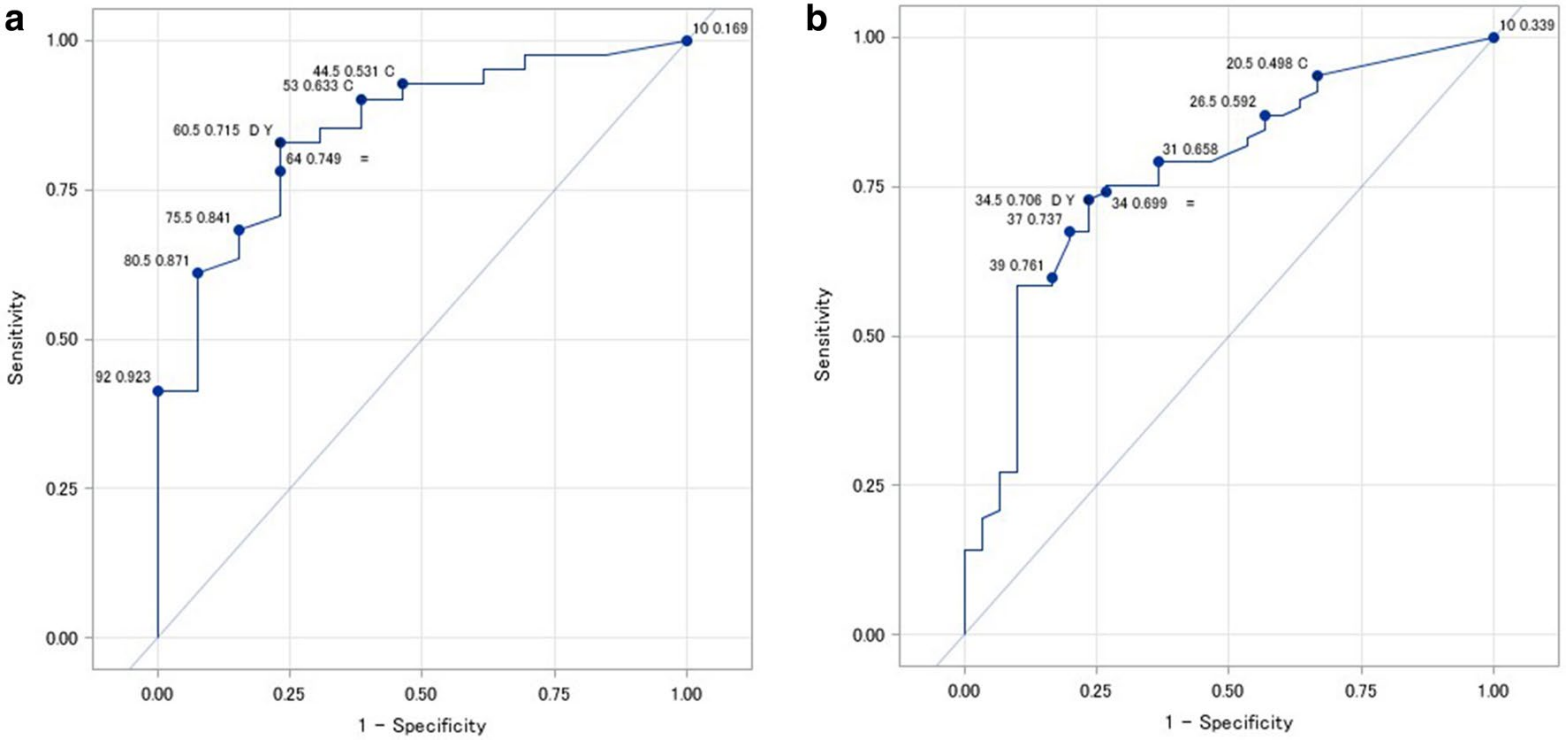
The receiver operating characteristic (ROC) curve was used to investigate the relationship of back muscle strength with sagittal malalignment (sagittal vertical axis [SVA] > 95 mm). Two methods to estimate Youden’s index (Y) and the distance from the top left corner of the receiver operating characteristic curve (D) were used to determine the cut-off value. The areas under the curve for male and female participants were 0.873 and 0.807, respectively. The cut-off values for male and female participants were 60 kg and 35 kg, respectively.

A total of 409 subjects with an average age of 73.5 years were analyzed in this study. The background characteristics of the participants are shown in Table 1. Subjects in the 40–95 and > 95 SVA groups were older (average age: 72.2 years in SVA < 40 vs. 74.5 years in SVA 40–95 vs. 78.4 years in SVA > 95; p < 0.001); had a greater sum of disc degeneration grade; and a greater number of OVF than those in the < 40 SVA group. There was no difference in sex, CCI, or SMI among the SVA groups. Osteoarthritis of both the hip and knee were seen more frequently in the high SVA group. The T score was lower in the high SVA group, whereas back, hip flexor, and knee extensor muscle strength was greater in the high SVA group. Based on the ROC curve analysis, back muscle strength showed moderate accuracy, while hip flexor and knee extensor strength showed low accuracy (i.e. AUC < 0.7). The cut-off value was 60 kg in male patients and 35 kg in female patients (AUC = 0.873 and 0.807, respectively) (Fig. 1). In the proportional odds model, odds ratios of age (OR = 1.11 per 1 year), disc degeneration (OR = 1.23 per 1 grade), and two or more vertebral fractures (OR = 2.20) showed a significant increase in the risk of SVA deterioration (Table 2), whereas stronger back, hip flexor, and knee extensor muscles reduced this risk (OR = 0.98, 0.94, and 0.96; p = 0.006, 0.036, and 0.021, respectively).

**Table 1:** Comparison of characteristics according to the sagittal vertical axis.

	SVA			p values
	< 40 N = 263	40–95 N = 101	> 95 N = 45	
Age (years)	72.2 (4.7)	74.5 (5.1)	78.4 (6.5)	< 0.001
Sex (female)	151 (61.6%)	63 (62.4%)	31 (68.9%)	0.274
BMI (kg/m^2^)	22.7 (2.9)	23.4 (3.6)	23.3 (4.4)	0.357
Charlson comorbidity index	0.6 (1.1)	0.6 (0.9)	0.5 (0.8)	0.939
**No. of vertebral fractures**				< 0.001
0	197 (74.9%)	65 (64.3%)	19 (42.2%)	
1	32 (12.2%)	20 (19.8%)	8 (17.8%)	
2-	34 (12.9%)	16 (15.8%)	18 (40.0%)	
T-score	− 0.59 (1.15)	− 0.85 (1.07)	− 1.24 (1.23)	0.001
Total disc degeneration	15 (5–20)	17 (7–20)	18 (11–20)	< 0.001
Trunk muscle mass (kg)	22.4 (3.7)	21.6 (3.8)	20.1 (4.0)	< 0.001
SMI (kg/m^2^)	7.04 (1.14)	7.04 (1.17)	6.86 (1.27)	0.662
Back muscle strength (kg)	65.7 (28.2)	54.2 (26.2)	32.5 (20.5)	< 0.001
Hip flexor strength (kg)	12.9 (4.7)	10.5 (4.2)	8.8 (4.0)	< 0.001
Knee extensor strength (kg)	18.8 (7.2)	17.3 (7.1)	12.4 (5.6)	< 0.001
Osteoarthritis of the hip (≥ K–L grade 2)	7 (2.7%)	4 (4.0%)	8 (17.8%)	< 0.001
Osteoarthritis of the knee (≥ K–L grade 2)	156 (59.3%)	78 (77.2%)	36 (80.0%)	< 0.001

*SVA* sagittal vertical axis, *BMI* body mass index, *SMI* skeletal muscle mass index, *K–L* Kellgren–Lawrence classification.

**Table 2:** Odds ratios obtained using the multivariate proportional odds model for the deterioration of sagittal vertical axis.

	Adjusted odds ratio	95% confidence interval		p values
Age (per a year)	1.12	1.07	1.18	< 0.001
Sex (male)	0.89	0.51	1.53	0.661
BMI (< 18.5 kg/m^2^)	1.51	0.63	3.61	0.360
BMI (> 25.0 kg/m^2^)	1,94	1.15	3.27	0.013
Charlson comorbidity index	0.87	0.70	1.09	0.231
Total disc degen. (per 1 grade)	1.17	1.08	1.26	0.007
**No. of vertebral fractures**				
1	1.74	0.96	3.16	0.068
≥ 2	2.35	1.34	4.11	0.003
Back muscle strength (per 1 kg)	0.98	0.97	0.995	0.009
Hip flexor strength (per 1 kg)	0.94	0.88	0.995	0.035
Knee extensor strength (per 1 kg)	0.96	0.93	0.999	0.044
Back muscle strength (male > 60 kg, female > 35 kg)	0.32	0.19	0.53	< 0.001
Osteoarthritis of the hip (≥ K–L grade 2)	3.22	1.21	8.56	0.019
Osteoarthritis of the knee (≥ K–L grade 2)	1.34	0.79	2.29	0.282

This model was adjusted for age, sex, body mass index, the total disc degeneration, back muscle strength (per 1 kg) or back muscle strength (> 60 kg in men, > 35 kg in women), hip flexor strength (per 1 kg), knee extensor strength (per 1 kg), and osteoarthritis of the hip and knee (≥ K–L grade 2).

**Figure 2:**
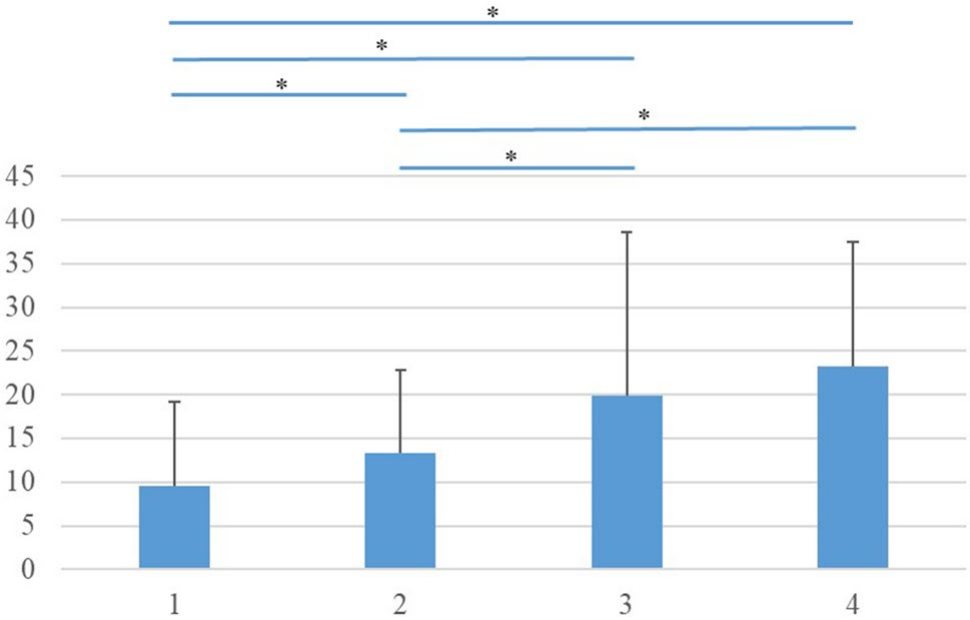
Oswestry Disability Index (ODI) among groups. There were significant differences between Groups 1 and 2, Groups 1 and 3, Groups 1 and 4, Groups 2 and 3, and Groups 2 and 4. p value < 0.001 by ANOVA. * < 0.05 according to Tukey test as post-hoc analysis. Group 1: PT < 20° and SVA < 40 mm; Group 2: PT ≥ 20° and SVA < 40 mm; Group 3: PT < 20° and SVA ≥ 40 mm; Group 4: PT ≥ 20° and SVA ≥ 40 mm.

Regarding the differences in ODI according to high/low PT and SVA, there were significant differences between Groups 1 and 2, Groups 1 and 3, Groups 1 and 4, Groups 2 and 3, and Groups 2 and 4 (Fig. 2). For analyzing the association of each muscle’s strength with compensatory mechanisms, 162 subjects with PI-LL mismatch > 10° were extracted. The multiple linear regression model was adjusted for age, sex, BMI, disc degeneration, vertebral fracture, PI, LL, back muscle strength, hip flexor strength, knee extensor strength, and TK or PT. The linear regression model for TK (R^2^ = 0.533) demonstrated a negative correlation with back muscle strength (r =  − 0.136, p = 0.003) (Table 3), whereas that for PT (R^2^ = 0.852) demonstrated a positive correlation with knee extensor strength (r = 0.125, p = 0.016) (Table 4).

**Table 3:** Association between thoracic kyphosis and subjects with PI-LL mismatch > 10° using a multivariate linear regression model.

	Coefficient	Standard error	p value	95% confidence interval	
Age (years)	0.272	0.174	0.120	− 0.071	0.615
Sex (female)	− 3.851	2.308	0.097	− 8.412	0.711
BMI (kg/m^2^)	0.236	0.241	0.330	− 0.241	0.712
Disc degeneration (per 1 grade)	− 0.185	0.309	0.551	− 0.795	0.426
Vertebral fracture (per 1 facture)	1.277	0.697	0.069	− 0.101	2.655
PI	− 1.333	0.141	< .001	− 1.611	− 1.055
PT	1.626	0.156	< .001	1.317	1.935
LL	− 1.050	0.089	< .001	− 1.226	− 0.874
Back muscle power (per 1 kg)	− 0.136	0.045	0.003	− 0.224	− 0.048
Hip flexor (per 1 kg)	− 0.343	0.217	0.116	− 0.772	0.086
Knee extensor (per 1 kg)	− 0.368	0.130	0.005	− 0.624	− 0.112
Osteoarthritis of the hip (per K–L 1 grade)	0.274	1.061	0.796	− 1.823	2.371
Osteoarthritis of the knee (per K–L 1 grade)	0.663	1.063	0.534	− 1.437	2.762

The linear regression model was adjusted for age, sex, body mass index, the total disc degeneration, PI, LL, PT, back muscle strength (per 1 kg), hip flexor strength (per 1 kg), knee extensor strength (per 1 kg) and osteoarthritis of the hip and knee (per K–L 1 grade).

**Table 4:** Association with pelvic tilt in patients with PI-LL mismatch > 10° using a multivariate linear regression model.

	Coefficient	Standard error	p value	95% confidence interval	
Age (years)	− 0.075	0.070	0.285	− 0.212	0.063
Sex (female)	0.219	0.931	0.814	− 1.621	2.059
BMI (kg/m^2^)	− 0.009	0.097	0.923	− 0.200	0.182
Disc degeneration (per 1 grade)	0.196	0.123	0.113	− 0.047	0.438
Vertebral fracture (per 1 facture)	0.307	0.281	0.276	− 0.248	0.862
PI	0.760	0.034	< .001	0.692	0.828
TK	0.260	0.025	< .001	0.210	0.309
LL	0.515	0.026	< .001	0.464	0.566
Back muscle power (per 1 kg)	0.019	0.018	0.295	− 0.017	0.055
Hip flexor (per 1 kg)	0.087	0.085	0.308	− 0.081	0.256
Knee extensor (per 1 kg)	0.125	0.051	0.016	0.024	0.226
Osteoarthritis of the hip (per K–L 1 grade)	0.058	0.424	0.891	− 0.780	0.896
Osteoarthritis of the knee (per K–L 1 grade)	− 0.307	0.424	0.471	− 1.146	0.532

The linear regression model was adjusted for age, sex, body mass index, the total disc degeneration, PI, LL, TK, back muscle strength (per 1 kg), hip flexor strength (per 1 kg), knee extensor strength (per 1 kg) and osteoarthritis of the hip and knee (per K–L 1 grade).

## Discussion

The findings of our study suggested that back muscle strength (especially, > 60 kg in men and > 35 kg in women), hip flexor strength, and knee extensor strength reduce the risk of spinal malalignment. Previous studies have reported an inverse correlation between back muscle strength and hyperkyphosis of the thoracic spine^18–20^. Sinaki et al. reported that back extensor strength correlates with the posture (i.e. the stronger the back extensors, the smaller the TK)^16^. Additionally, Imagama et al. demonstrated that maintenance of spinal sagittal alignment was related to muscle strength, and these factors stabilized body balance and were mutually related^26^. Therefore, improvement of physical abilities through muscle training might be important, not only to maintain spinal sagittal alignment but also to prevent falls and maintain activities of daily living in the elderly population^26^. Feng et al.^27^ conducted a single-blind, randomized controlled trial on students with a thoracic kyphosis angle > 40° measured using the Spinal Mouse; they found that a corrective functional exercise program improved the exaggerated TK and range of motion in young subjects^27^. Hongo et al. demonstrated that back extensor strength was significantly associated with LL, indicating the potential importance of strengthening the back extensor for improving or maintaining LL^28^. They also conducted a randomized controlled study that suggested that low-intensity, back-strengthening exercises were effective in improving the QOL and back extensor strength in patients with osteoporosis^29^. These findings indicate that improvement of back muscle strength helps maintain LL and reduce TK, which are linked to improvement in spinal sagittal alignment and QOL.

Our study demonstrated that osteoporosis and disc degeneration were important factors in global spinal alignment. Fechtenbaum et al. demonstrated, using multivariate analysis, that parameters that were significantly associated with abnormal spinal balance were the presence of at least one vertebral fracture [odds ratio (OR) = 4.96, p < 0.0001], age (OR = 1.07, p = 0.0006), and high PI as a protective factor (OR = 0.93, p < 0.0001)^10^. Sinaki et al. demonstrated the long-term effect of strong back muscles on the reduction of vertebral fractures in women^30^. However, global spinal alignment can be abnormal even in subjects without vertebral fractures^10^. Mika et al. also suggested that decreased bone mineral density influences TK; however, despite decreased bone mineral density, if the back muscles are sufficiently strong, spinal deformities do not occur^18^. This might reflect the association of osteoporosis with disc degeneration. An animal study revealed that estrogen deficiency exacerbated intervertebral disc degeneration induced by spinal instability, whereas estrogen supplementation alleviated the progression of disc degeneration related to osteoporosis^31^. Wei et al. demonstrated that the SVA value is positively correlated with the overall degree of lumbar disc degeneration, which corroborates our results^32^.

PT allows patients to achieve sagittal balance in the setting of decreased LL, with the primary compensatory mechanisms being hip extension and knee flexion. Lafage et al. confirmed that pelvic position measured via PT correlates with disability and QOL^4^. Furthermore, our data showed that there was a significant difference in subjects with high PT and low SVA versus those with high PT and high SVA. This indicated that compensatory mechanisms play an important role in subjects with deteriorated spinopelvic harmony.

This study was the first to reveal the association of muscle strength, including strength of the lower extremities, with compensatory mechanisms. Diebo et al. demonstrated that, contrary to the first two mechanisms (TK and PT), the values of pelvic shift and knee flexion increase with progressive deformity, along with an increase in their percentage of contribution^33^. Therefore, with increasing mismatch between PI and LL, the pelvic version becomes exhausted, at which point there is a steady transfer of compensation toward the lower limbs and pelvic translation posteriorly. The quadriceps femoris is the largest muscle for knee extension. Muscle strength might be necessary to maintain the knee flexion position^34^. Increasing PT is a limited compensatory mechanism used to maintain normal sagittal balance in the setting of PI-LL mismatch. The pelvic capacity to participate in spinopelvic compensation by tilting remains unclear. However, the pelvis cannot tilt more than the PI value, because the sacral slope cannot be a negative value in the standing position^35^.

In our study, the hip flexor was found to be important for sagittal malalignment but not for compensatory mechanisms. The psoas major muscle was the primary flexor of the hip joint. Xia et al. demonstrated that there was no relationship between PT and the psoas muscle^36^; whereas Menezes-Reis et al. demonstrated that SVA was inversely correlated with the psoas volume^37^. Other studies have demonstrated that the magnitude of the multifidus and psoas muscle was negatively associated with the presence of a marked deformity^38,39^. Santaguida et al. reported that the mechanical action of the psoas major did not change as a function of lumbar spine lordosis because the muscle path of action changes in accordance with changes in the spine posture^40^. The psoas major primarily functions not only as a hip flexor but also as a stabilizer of the lumbar spine^41^, thereby playing an important role in maintaining economic posture.

There were several limitations in this study. First, hip extensors, which might be very important to PT, were not evaluated. A significant decrease in the level of hamstring stiffness was recorded in the experimental group, accompanied by an increase in anterior PT^42^. Frigo et al. suggested co-contraction of the quadriceps and hamstrings as a strategy to increase the hip extension function of the hamstrings^42^. Any potential role of the hamstrings appears to be secondary to their role as concentric, isometric, and eccentric static and dynamic stabilizers of the trunk and pelvis^34^. Second, this study was conducted as a cross-sectional study. Therefore, it is unclear whether improvement of the knee extensors and back muscle strength affects the compensatory mechanism to maintain sagittal alignment. However, this study deepens our understanding of the underlying mechanism that contributes to adult spinal deformity, which might help to devise targeted treatment strategies including the muscles. Third, motor-neuron disease that might affect posture and muscle strength was not evaluated. However, there were no subjects with Parkinson disease which was a disorder of the central nervous system with postural instability. Finally, alignment of the lower limbs including femur obliquity angle and knee flexion angle was not evaluated.

In conclusion, this study investigated the relationship between muscle strength and compensatory mechanisms. Back muscle, hip flexor, and knee extensor strength was associated with sagittal spinal alignment. Back muscle strength was important to decrease TK, and knee extensor strength was associated with PT.
